# 18β-glycyrrhetinic Acid Modulated Autophagy is Cytotoxic to Breast Cancer Cells

**DOI:** 10.7150/ijms.80302

**Published:** 2023-02-13

**Authors:** Yu-Chih Hsu, Wen-Che Hsieh, Shu-Hsin Chen, Yi-Zhen Li, Hui-Fen Liao, Mei-Yi Lin, Shew-Meei Sheu

**Affiliations:** 1 Department of Chinese Medicine, Ditmanson Medical Foundation Chia-Yi Christian Hospital, Chia-Yi City 60002, Taiwan; 2 Department of Chinese Medicine, China Medical University Hospital, Taichung City 40447, Taiwan; 3 Department of Medical Research, Ditmanson Medical Foundation Chia-Yi Christian Hospital, Chia-Yi City 60002, Taiwan; 4 Department of Biochemical Science and Technology, National Chiayi University, Chia-Yi City 60004, Taiwan; 5 Department of Food Nutrition and Health Biotechnology, Asian University, Taichung City 41354, Taiwan

**Keywords:** 18β-glycyrrhetinic acid, autophagy, apoptosis

## Abstract

The development of endocrine therapy resistance in the luminal A subtype of breast cancer is related to the appearance of protective autophagy. The bioactive component from the root of licorice, 18β-glycyrrhetinic acid (18β-GA), has many antitumor properties. Whether 18β-GA can modulate autophagy to inhibit proliferation of the luminal A subtype is still unclear. The proportion of apoptosis caused by 18β-GA in MCF-7 and T-47D cells was determined using flow cytometry. The autophagy marker, LC3-II conversion, was investigated using Western blotting, and a Premo^TM^ Tandem Autophagy Sensor Kit. We found that the concentration (150-μM) of 18β-GA caused caspase-dependent apoptosis and LC3-II accumulation or blocked autophagic flux. Moreover, 18β-GA-mediated apoptosis was improved using rapamycin but reversed by 3-methyladenine (3-MA) addition. The phosphorylation level of Jun-amino-terminal kinase (JNK) was increased significantly in the 18β-GA treatment and combined incubation using rapamycin. A JNK inhibitor (SP600125) significantly inhibited 18β-GA-mediated apoptosis, LC3-II accumulation and rescued the numbers of MCF-7 and T-47D colony formation. Especially, 18β-GA can inhibit xenograft tumor growth in BALB/c nude mice. These data indicate the combination of 18β-GA with rapamycin or 3-MA can sensitize or decrease MCF-7 and T-47D cells to 18β-GA-induced apoptosis, respectively. 18β-GA modulated autophagy is cytotoxic to luminal A subtype breast cancer cells through apoptosis promotion and JNK activation.

## Introduction

Breast cancer is the most frequently occurring malignancy and the leading cause of cancer-associated death in women worldwide 1. The World Health Organization reported that 2.3 million women were diagnosed with breast cancer globally, causing 685,000 deaths in 2020. Breast cancer is a heterogeneous disease with varying intrinsic genetic subtypes. Surgery, drug treatment, and radiotherapy are the principal therapies used to treat breast cancer 2. Treatment challenges include drug resistance and diverse efficacy and the benefit of chemotherapy in the intrinsic subtypes.

According to immunohistochemistry markers, such as estrogen receptor (ER), progesterone receptor (PR), human epidermal growth receptor 2 (HER2), and Ki-67 (a marker of cell proliferation), breast tumors can be approximately classified into four subtypes. Luminal A (ER+/PR+/HER2-/Ki-67 low) is the most common subtype (50%-60% of all breast cancers) 3. A retrospective multicenter study indicated that adjuvant chemotherapy failed to enhance the recurrence-free rate of patients with nodal-positive luminal A breast cancer 4. Some patients eventually develop endocrine therapy resistance, associated with protective autophagy 5.

Chinese medicine has been proven to be a promising cancer adjuvant treatment for lung cancer, colorectal cancer, and breast cancer 6-8. The roots and rhizomes of the *Glycyrrhiza* species (licorice) have been used extensively in herbal medicine and natural sweeteners for thousands of years in Chinese and European areas. Numerous studies on licorice have proposed its possible pharmacological qualities related to anti-inflammatory, antiviral, antimicrobial, antioxidant, and antitumor activities 9. An important component is its glycyrrhizic acid (glycyrrhizin, GL), which is converted to 18β-glycyrrhetinic acid (GA) by human intestinal normal flora.

18β-GA shows multiple biological functions, including anti-proliferative, pro-apoptotic, anti-invasive, and anti-metastatic activities 10-12. 18β-GA causes protective autophagy in hepatocellular carcinoma cells 13, 14, but blocks autophagy to enhance the apoptosis in human sarcoma cells 15. It remained unclear whether 18β-GA's effect on autophagy is protective or cytotoxic for breast cancer and the interaction between apoptosis and autophagy in the survival of breast cancer.

Autophagy plays a crucial role in the pro-survival or pro-death of breast cancer cells and is considered a therapeutic target 16, 17. Inhibition of autophagy can increase the possibility of re-sensitizing previously antiestrogen-resistant breast cancer cells 5, showing that modulated autophagy is important in treating of ER+ breast cancer cells. In this study, the mechanism of 18β-GA-inhibited proliferation of breast cancer cells (luminal A: MCF-7 and T-47D), including cell cycle arrest, apoptosis, and autophagy-mediated apoptosis, were explored.

## Materials and Methods

### Breast cancer cell lines and culture

Human breast cancer cell lines, ER-positive luminal A (MCF-7 and T-47D), were bought from the Bioresource Collection and Research Center, Taiwan. MCF-7 was cultured in RPMI1640 medium (Gibco BRL, Grand Island, NY, USA) supplemented with 8% fetal bovine serum (FBS). T-47D was maintained in RPMI1640 medium using 8% FBS, containing 1.5-g/L sodium bicarbonate, 4.5-g/L glucose, 10-mM HEPES, and 1.0-mM sodium pyruvate and 0.2 I.U. bovine insulin per ml. The cell lines were kept in a humidified atmosphere with 5% CO_2_ at 37˚C.

### Reagents and antibodies

The reagent, 18β-GA, was bought from Sigma-Aldrich (St. Louis, MO, USA). The pan-caspase inhibitor, Z-VAD-FMK and 3-methyladenine (3-MA) were collected from Adooq Bioscience (Irvine, CA, USA). Rapamycin was acquired from LC Laboratories (Woburn, MA, USA). Antibody associated with the apoptosis against poly (ADP-ribose) polymerase (PARP) (#9542) and antibodies targeting phospho-SAPK/JNK (Thr183/Tyr185) (#4668), and JNK2 (#9258) were acquired from Cell Signaling Technology, Inc. (Beverly, MA, USA). A rabbit polyclonal antibody against the autophagosomal marker protein, microtubule-associated protein 1 light chain 3 (LC3) was bought from Abcepta, Inc. (San Diego, CA, USA) for Western blotting. A mouse monoclonal antibody against sequestosome 1 protein, also designated p62, was acquired from Santa Cruz Biotechnology (Santa Cruz, CA, USA). Anti-glyceraldehyde-3-phosphate dehydrogenase (GAPDH) monoclonal antibody was bought from Taiclone (Taipei, Taiwan).

### Viability assay

Cells were seeded in 96-well microplates at a density of 5×10^3^ cells/well overnight. After the cells were treated using a medium containing varying concentrations of 18β-GA (0, 50, 100, 150, and 200-μM) for 24 and 48 h, a Cell Counting Kit-8 (Sigma-Aldrich) was used to evaluate the cell viability. The cells were incubated with WST-8 solution for two hours, then absorbance at 450-nm and reference at 655-nm were recorded with a Model 680 microplate reader (Bio-Rad Laboratories, Inc., Hercules, CA, USA). The percentage of viable cells was calculated using the formula: A_treated_/A_control_.

### Colony formation assay

Cells (2×10^3^ /well) were seeded in 12-well plates and treated using the indicated concentrations of 18β-GA or combined with JNK inhibitor (5-μM). The medium was substituted with fresh culture medium every three days. After 6-15 days, the colonies were stained using 1% Crystal violet (Sigma-Aldrich) and photographed. Counting the colonies was conducted using the Alpha Innotech Imaging system (Alphatron Asia Pte Ltd, Singapore).

For LC3-II puncta images, 2×10^3^ cells were plated in an α plus^®^ 35*12-mm glass dish (Alpha Plus Scientific Corp, Taoyuan, Taiwan) overnight. After pre-treatment with or without JNK inhibitor (5-μM) for two hours, 18β-GA (80-μM) was added for incubation in the presence or absence of JNK inhibitor. A fresh culture medium with the same concentration of reagents was used to alter the previous medium every three days. After incubation for 6-15 days, cells were fixed using 4% paraformaldehyde (Sigma-Aldrich) at room temperature (RT) for ten minutes. Next, cells were incubated using cold acetone for three minutes at 4ºC. Hydrogen peroxide (3%, Acros Organics, Geel, Belgium) was applied for five minutes at RT. Then, incubation with anti-LC3 antibody (PM036, 1:200, *MBL International Corporation*, Woburn, MA, USA) was conducted overnight at 4ºC. Alexa Fluor^TM^ 488 goat anti-rabbit IgG (H+L) antibody (1:500, Invitrogen, Carlsbad, CA, USA) was used to label LC3-II at RT for one hour. Finally, cells were stained using 300-nM of DAPI (Sigma-Aldrich). Between each step of the treatment, cells were washed thrice with 1× phosphate-buffered saline (PBS) and maintained in 1× PBS during the investigation using a confocal microscopy LSM800 (Carl Zeiss Microscopy GmbH, Jena, Germany).

### Cell cycle and apoptosis assays

For cell cycle analysis, 1×10^6^ cells were adhered to a 10-cm dish overnight and underwent serum starvation for 24 h. Cells were treated using solvent (0.1% DMSO) or 18β-GA for 24 and 48 h before being detached with trypsin and centrifuged at 2000 rpm for five minutes. Then, cell pellets resuspended in 1× PBS (1-ml) were fixed with methanol (2-ml) and stored at 4˚C. After washing with 1× PBS, cells were stained using 40-µg/ml propidium iodide (PI) (Sigma-Aldrich) containing RNAase (50-µg/ml) for 30 min in the dark at RT. Cell cycle distribution was evaluated using a FACSCan flow cytometer (Becton Dickinson, San Diego, CA, USA). DNA content was further assessed using Modfit LT 3.3 software. For the apoptosis analysis, 3×10^5^ cells were plated in a 6-cm dish overnight and further incubated with and without 18β-GA (150-μM) for 48 h. The apoptotic cells were labeled using the Annexin V-Fluorescein Isothiocynate (FITC) Apoptosis Detection Kit (Strong Biotech, Taipei, Taiwan) and PI, which were further analyzed using a FACSCan flow cytometer.

### Monitoring autophagy with the RFP-GFP-LC3B Kit

Cells (1×10^4^) were seeded in an α plus^®^ 35*12-mm glass dish (Alpha Plus Scientific Corp) and allowed to adhere overnight. The autophagy progress was monitored using the Premo^TM^ Autophagy Tandem Sensor red fluorescent protein (RFP)-green fluorescent protein (GFP)-LC3B Kit (Life Technologies, Carlsbad, CA, USA), according to the manufacturer's guidelines. Treatment using 4.5-μl BacMam reagent containing RFP-GFP-LC3B DNA was conducted overnight, and specified concentrations of 18β-GA were subsequently added for 24 h. The positive control used rapamycin (30-μM) to cause autophagic flux. The live cell imaging solution containing Hoechst 33342 (1-μg/mL), was added and incubated for 20 min in the dark. After washing the cells using 1× PBS, images were obtained using a Zeiss laser scanning confocal microscope LSM800 (Carl Zeiss Microscopy GmbH).

### Western blotting

Cells were lysed with the M-PERTM mammalian protein extraction reagent (Thermo Fisher Scientific Inc., Rockford, IL, USA), containing a 0.1% protease inhibitor cocktail. Total protein (40-μg) of each sample was equally loaded, separated on 8% or 12% of sodium dodecyl sulfate-polyacrylamide gel electrophoresis (SDS-PAGE) gels, and transferred to polyvinylidene fluoride membranes. Pro-PARP, cleaved PARP, and p-JNK were separated on 8% of SDS-PAGE gels, but 12% of SDS-PAGE gels were used to separate the other target proteins, such as LC3-II, p62, and JNK. To identify the target proteins using primary antibodies (1:1000) at 4°C for overnight incubation and horseradish peroxidase-conjugated secondary antibodies (1:10000) at RT for one hour, the band signals were measured using Immobilon Western Chemiluminescent HRP Substrate (EMD Millipore Corporation, Billerica, MA, USA), and found using a BioSpectrum^®^ imaging system (UVP). Antibodies against PARP were used to confirm apoptosis, and autophagy was identified using anti-LC3-II and p62 antibodies. Antibodies of total and phosphor-JNK were applied to detect signal pathway-mediated cell death.

### Xenograft model in BALB/c nude mice

Animal experiments were conducted following protocols approved by the Institutional Animal Ethical Committee of National Chiayi University (IACUC Approval No: 111028). The 5-week-old female BALB/c nude mice were bought from BioLASCO Taiwan Co., Ltd (Taipei, Taiwan) and acclimated at the Animal Facility of National Chiayi University for one week. MCF-7 cells (8×10^6^) suspended in 1× PBS and 50-μl matrigel (1:1) (CLS354234, Merck KGaA, Darmstadt, Germany) were implanted subcutaneously into the left flank of the nude mice on Day zero, and mice were randomly divided into two groups (four mice per group). Control group mice were intraperitoneal (i.p.) injected with DMSO and 18β-GA (25-mg/kg) was administered to the experimental group mice on Day 5. Every 2-3 days, each mouse was i.p. ingestion with DMSO or the 18β-GA dosage. The body weight of mice was measured every time before drug injection. Closing the experiment on Day 17, the nude mice were sacrificed, and the tumors were harvested and weighed. Liver tissues were fixed using 10% formalin, and paraffin sections were deparaffined, rehydrated, and stained using hematoxylin and eosin. These sections were evaluated using light microscopy.

### Statistical analysis

The data indicated in this study are presented as the mean ± standard deviation (SD). Statistical significance was determined using one-way ANOVA with post hoc test and Bonferroni correction to compare multiple groups and with an independent t-test to compare two groups using SPSS (Windows version 21), and *p*-values of **<** 0.05 were considered statistically significant.

## Results

### 18β-GA inhibited breast cancer cell proliferation* in vitro*

**Figure 1:**
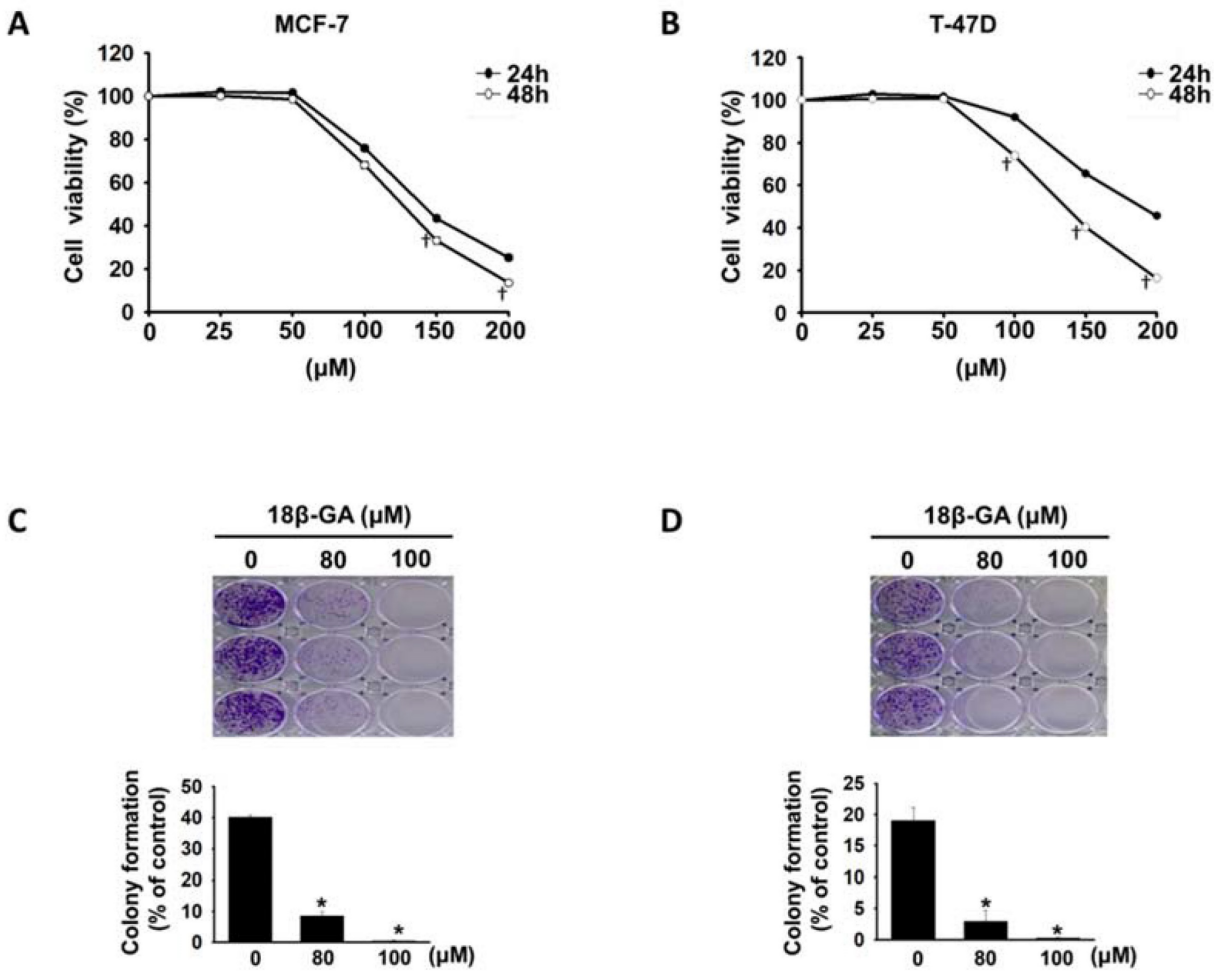
** Anti-proliferation of 18β-GA on luminal A breast cancer cells.** MCF-7 (A) and T-47D (B) cells were treated using 18β-GA ranging from 25-200-μM for 24 and 48 h. Cell viability was detected using the CCK-8 assay. † indicate a significant difference (*p* < 0.05) comparing the effect of 18β-GA treatment on cell growth at 24 h with that at 48 h by independent t test. Colony formation assays were conducted on MCF-7 (C) and T-47D (D) cells treated with 80 and 100-μM 18β-GA for 8-15 days. Visible colonies were stained using crystal violet. The control group contained 0.1% DMSO. Data are indicated as the mean ± SD from three independent experiments. Significance was analyzed by one-way ANOVA followed by the Bonferroni test (*p* < 0.05). * shows a significant difference compared with the DMSO control.

18β-GA did not affect cell proliferation of immortalized normal mammary epithelial cell line (MCF-10A) 12. To evaluate the inhibitory effect of 18β-GA on luminal A breast cancer cells, MCF-7 and T-47D cells were treated with 18β-GA at concentrations ranging from 25-200-μM. As indicated in Figs. 1A and 1B, 18β-GA decreased the viability of MCF-7 and T-47D cells in a concentration- and time-dependent manner. The IC_50_ values of 18β-GA for MCF-7 and T-47D were 125.8 and 135.6-μM at 48 h. To test the effect of 18β-GA on the tumorigenicity of breast cancer cells, colony formation assays were performed. Also, 18β-GA dose-dependently reduced the colony numbers of MCF-7 and T-47D cell lines (Figs. 1C and 1D). These data indicate that 18β-GA dose-dependently inhibits breast cancer cell proliferation *in vitro.*

### 18β-GA induced cell cycle G0/G1 arrest and apoptosis

**Figure 2:**
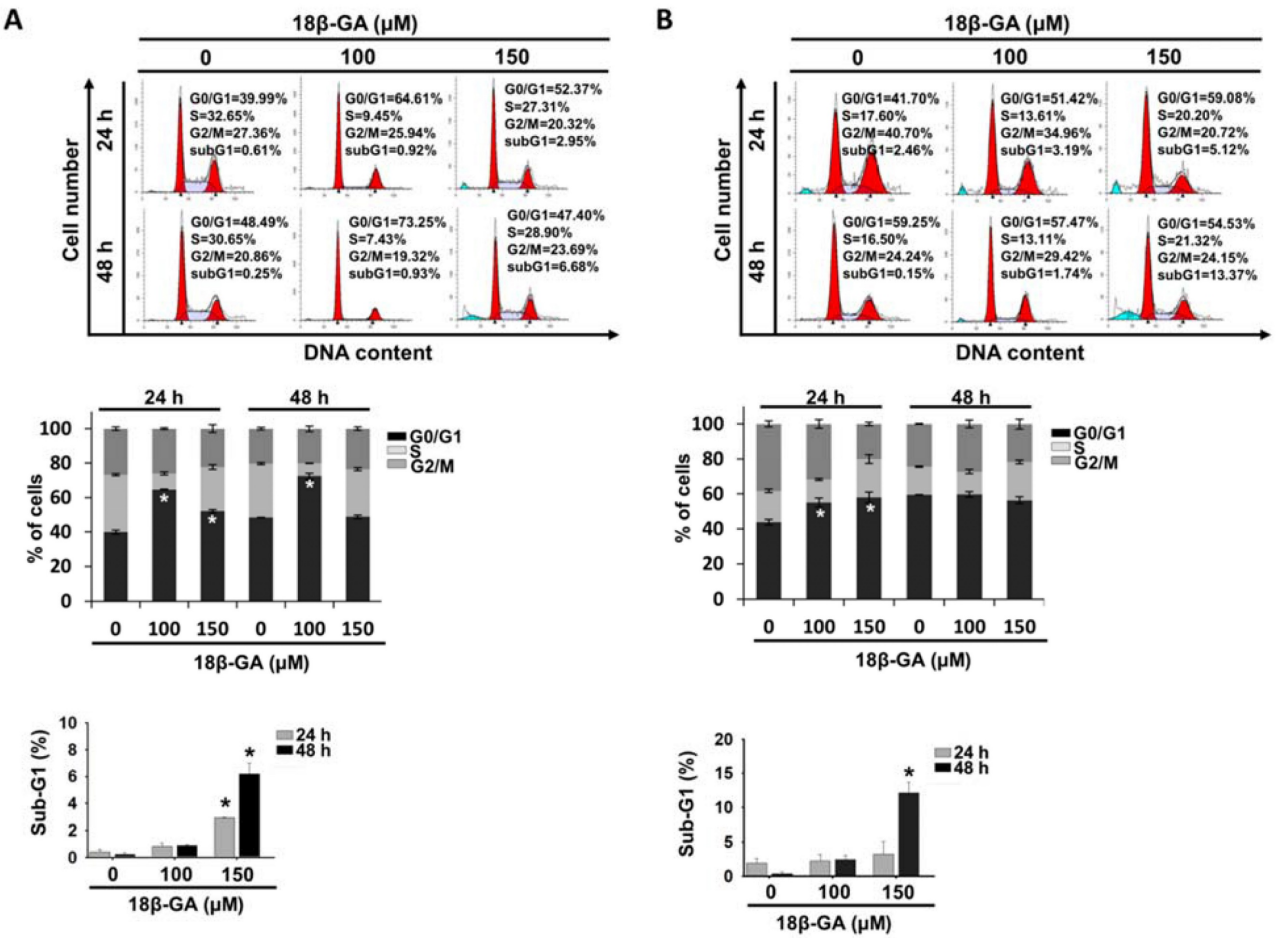
** 18β-GA caused G0/G1 cell cycle arrest.** MCF-7 (A) and T-47D (B) were incubated using the indicated concentrations of 18β-GA for 24 and 48 h. The distribution of the cell cycle was detected using flow cytometry. The graphs display one representative data point of the cell cycle distribution. The mean percentage of each cell cycle phase acquired from three individual experiments is indicated in the histogram. The subG1 proportion of cells treated using the indicated concentration of 18β-GA for different periods are presented in the other histogram. * shows a significant difference (*p* < 0.05) compared to the percentage of the DMSO control, as analyzed using one-way ANOVA followed by the Bonferroni test.

To explore the possible inhibition mechanism of cell proliferation, the influence of 18β-GA on the cell cycle and apoptosis was examined using flow cytometry. It was found that 100-μM 18β-GA induced G0/G1 arrest in MCF-7 cells at 24 and 48 h (Fig. 2A), whereas a higher concentration (150-μM) did not obviously arrest the cell cycle at 48 h. In Fig. 2B, incubation with increasing 18β-GA concentrations induced accumulation of the number of T-47D cells in the G0/G1 phase for 24 h, but not for 48 h. There were significant sub-G1 accumulations when a higher concentration (150-μM) of 18β-GA was applied to both cell types after 24 and/or 48 h of treatment (Figs. 2A and 2B).

**Figure 3:**
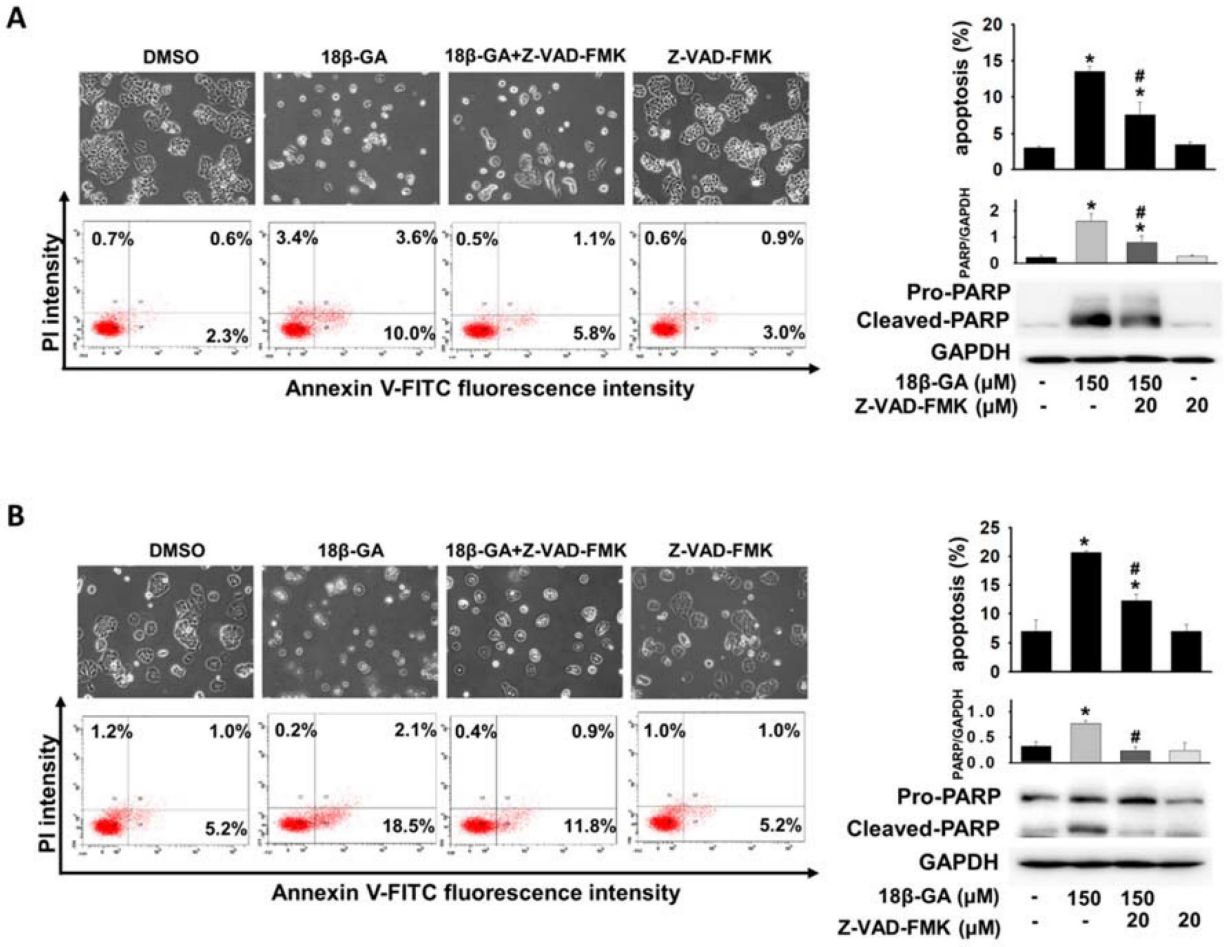
** 18β-GA induced apoptosis in breast cancer cells.** MCF-7 (A) and T-47D (B) were treated with 18β-GA (150-μM) for 48 h. Apoptosis induced by 18β-GA was stained using Annexin V-FITC/PI fluorescence and examined using flow cytometry. The cell morphology and dual parameter dot plot of FITC (x-axis) versus PI (y-axis) were based on one representative data point of three independent experiments with similar results. Three individual apoptosis experiments for both cell lines were analyzed, and the results are presented as the mean ± SD. Pan-caspase inhibitor (Z-VAD-FMK) was used to inhibit caspase-dependent apoptosis, which was verified using Western blotting with the reduction of cleaved-PARP. The expression of cleaved PARP related to GAPDH was calculated and shown in the histogram. The percentage of 18β-GA-induced apoptosis in the absence or presence of Z-VAD-FMK was also analyzed. *Shows a significant difference compared with the cell control, and # shows a significant difference compared with the 18β-GA treatment, as analyzed using one-way ANOVA followed by the Bonferroni test.

Annexin V-FITC/PI staining was used to verify whether 18β-GA induced cell death through apoptosis. Flow cytometric analysis indicated that 150-μM 18β-GA caused a significant increase in the apoptotic population compared with MCF-7 and T-47D control cells (Figs. 3A and 3B). Using a pan-caspase inhibitor to prove the involvement of the caspase-dependent pathway in 18β-GA-induced apoptosis, Z-VAD-FMK significantly suppressed 18β-GA-induced apoptosis and the cleavage of pro-PARP (Figs. 3A and 3B).

### 18β-GA blocked autophagic flux

**Figure 4:**
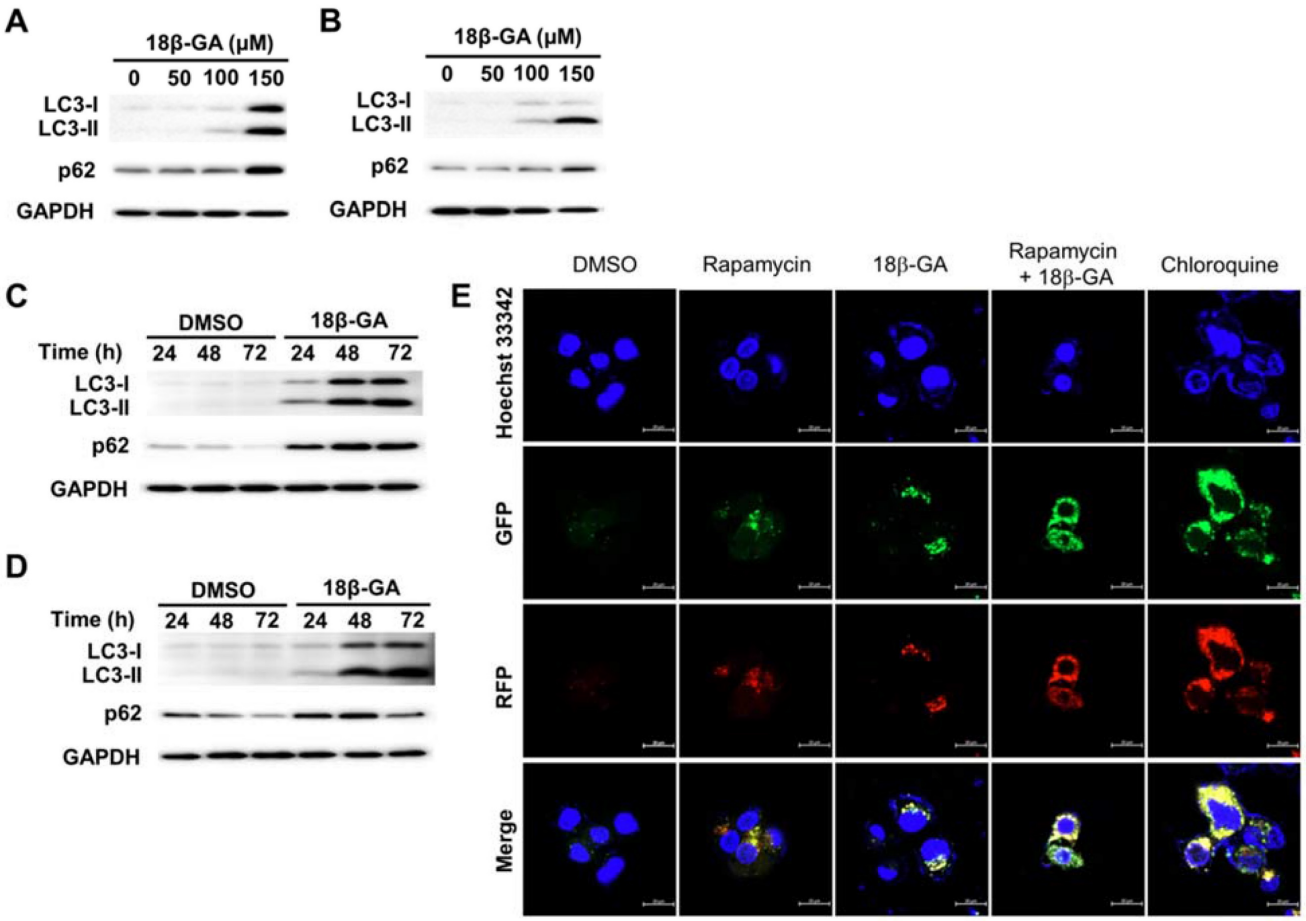
** 18β-GA increased the conversion of LC3-II in a dose- and time-dependent manner.** MCF-7 (A, C) and T-47D (B, D) cells were treated using the indicated concentrations of 18β-GA for 24 h or incubated with 150-μM for 24, 48, and 72 h. The autophagy markers LC3-II and p62 were identified using Western blotting. GAPDH was used as the loading control. (E) 18β-GA blocked autophagic flux. MCF-7 cells were transduced with tandem RFP-GFP-LC3B to monitor the fluorescent images of autophagosomes and autophagolysosomes. The RFP-GFP-LC3B expressing cells were treated using chloroquine (50-μM), rapamycin (30-μM), 18β-GA (150-μM), or a combination of the two drugs. In the merged images, yellow or orange puncta (RFP-GFP-LC3B) show the autophagosomes, whereas red puncta (RFP-LC3B) is preserved in autophagolysosomes because the acidification of lysosomes quenches the green fluorescence. Images were obtained using a 63× oil immersion objective. The scale bar is 20-μm.

Autophagy is involved in the survival of breast cancer cells 16. Cytosolic LC3-I conjugates phosphatidylethanolamine to form LC3-II. LC3-II is recruited to the autophagic vacuole membranes as a specific marker for autophagy. It was found that 18β-GA induced dose-dependent accumulation of LC3-II and p62 in MCF-7 and T-47D cells (Figs. 4A and 4B). Upon 18β-GA treatment at 150-μM, the increase in LC3-II conversion occurred time-dependently (Figs. 4C and 4D). To monitor the autophagy stage that 18β-GA interfered with, a Premo^TM^ Tandem Autophagy Sensor Kit was used to image the fusion process of autophagosomes and lysosomes to form autolysosomes. In the autophagosomes (neutral pH), the LC3-II fused with a GFP and RFP was green and red-positive, and showed yellow/orange puncta. The fluorescence of GFP was pH-sensitive and quenched in the acidity of the autolysosomes to appear as red puncta. In Fig. 4E, chloroquine (CQ) was applied to arrest endogenous autophagic flux by impairing lysosomal function, causing LC3-II accumulation in autophagosomes (yellow/orange). MCF-7 cells treated using 18β-GA also accumulated LC3-II in autophagosomes, similar to the response of CQ. Moreover, 18β-GA further suppressed rapamycin-induced autophagic flux, indicating autophagosome accumulation. These results showed that 18β-GA can block autophagic flux.

### Autophagy induction enhanced 18β-GA-induced apoptosis

**Figure 5:**
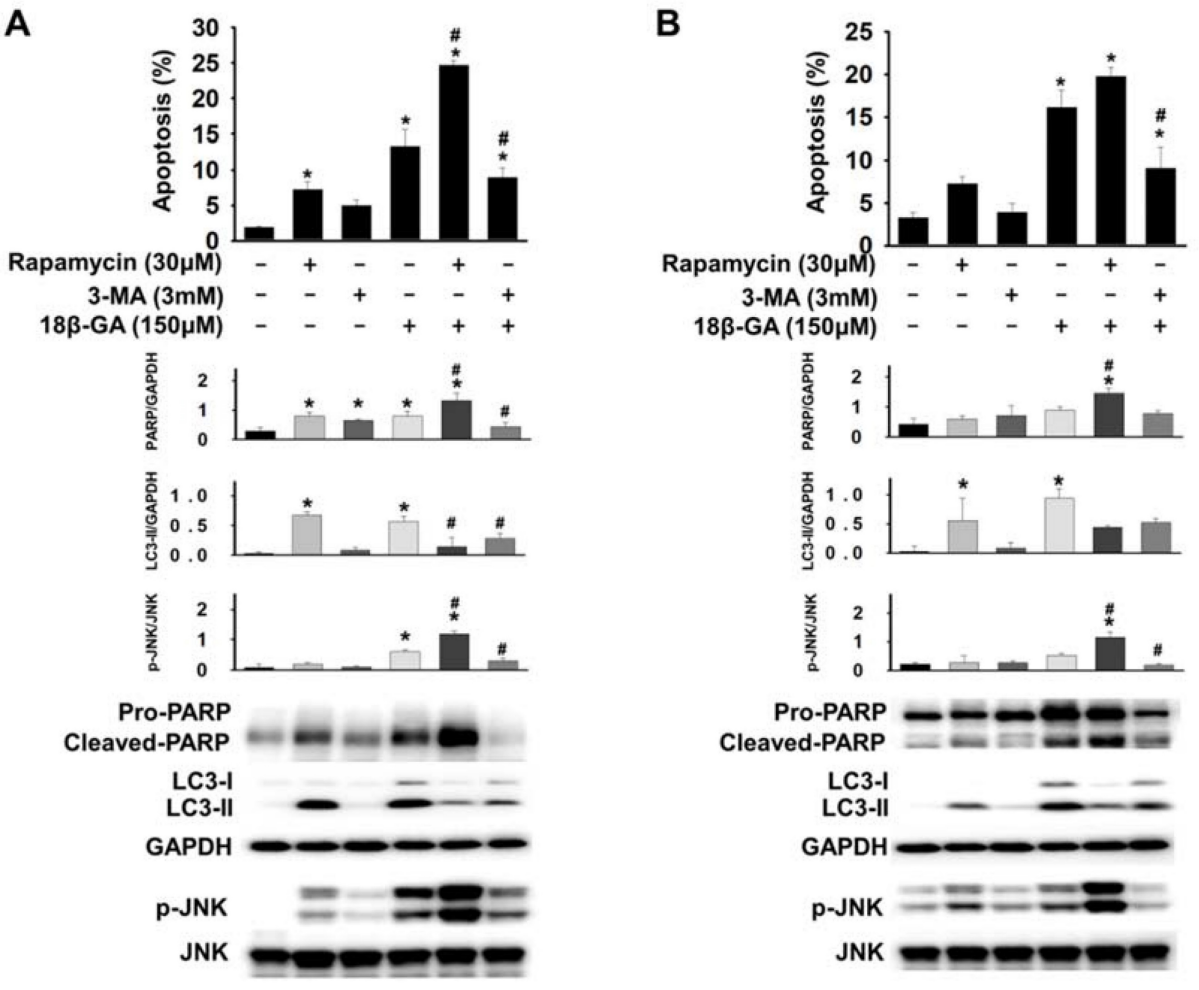
** Autophagy induction promoted 18β-GA-induced apoptosis.** MCF-7 (A) and T-47D (B) cells were pre-treated with or without rapamycin or 3-MA for two hours, and then incubated with 150-μM 18β-GA in the absence or presence of rapamycin and 3-MA for 48 h. Apoptosis was examined using flow cytometry. The cell lysates were obtained after 24 h for Western blotting. Target protein related to GAPDH and the corresponding ratio of p-JNK versus total JNK are shown in histograms. * shows a significant difference (*p* < 0.05) compared to the cell control, and # indicates a significant difference compared to the 18β-GA treatment, as assessed using one-way ANOVA followed by the Bonferroni test.

To determine whether 18β-GA-modulated autophagic flux was cytoprotective or cytotoxic, cells pre-treated using rapamycin (autophagy inducer) or 3-MA (initiated autophagy blocker) were further incubated using 18β-GA. A combined treatment using rapamycin enhanced 18β-GA-mediated apoptosis in MCF-7 and T-47D cells (Figs. 5A and 5B), whereas treatment using 3-MA significantly decreased the apoptosis resulting from the 18β-GA induction. In Western blotting, treatment with 18β-GA elevated LC3-II, and the apoptosis marker cleaved PARP. The addition of 3-MA to suppress autophagy initiation decreased 18β-GA-caused LC3-II accumulation and the cleavage of pro-PARP. The combined treatment with rapamycin and 18β-GA enhanced the cleavage of pro-PARP as compared with 18β-GA alone. However, LC3-II in this situation was reduced, possibly because of the elevation of cellular apoptosis. Collectively, these data showed that 18β-GA-modulated autophagy is cytotoxic by promoting apoptosis.

**Figure 6:**
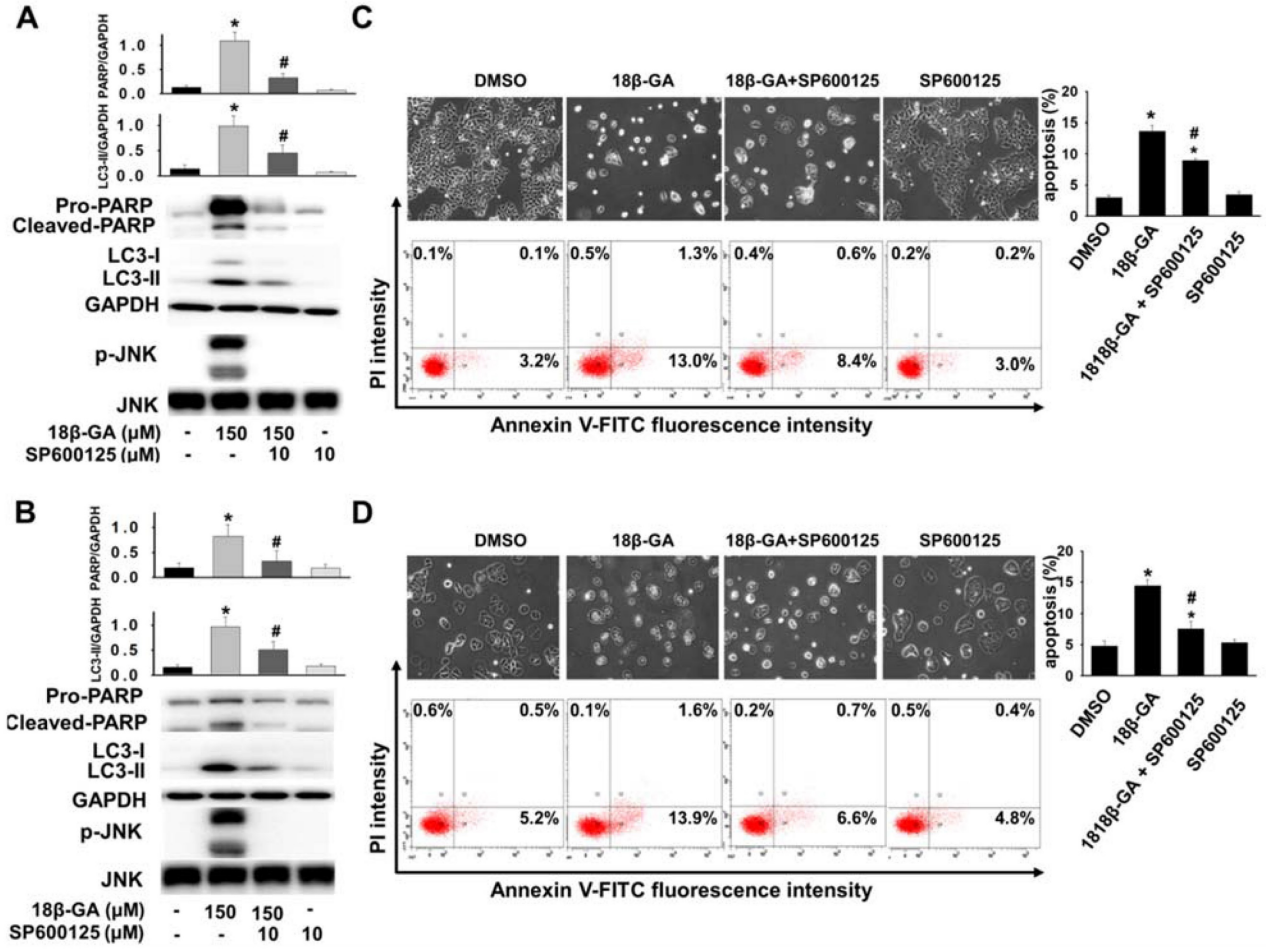
** Inhibition of p-JNK reduced 18β-GA-induced apoptosis and LC3-II.** MCF-7 (A, C) and T-47D (B, D) cells were pre-treated with or without JNK inhibitor (SP600125) for two hours. Then, 18β-GA was further added to both cells for incubation for 48 h. Western blotting detected the protein expression of cleaved PARP, LC3 conversion, p-JNK, JNK, and GAPDH, which was expressed by the ratio of the target protein related to GAPDH or total JNK. AnnexinV-FITC/PI double staining was used to detect the apoptosis percentage. * indicates a significant difference (*p* < 0.05) compared with the percentage of the cell control, and # shows a significant difference compared with the 18β-GA treatment, as assessed using one-way ANOVA followed by the Bonferroni test.

**Figure 7:**
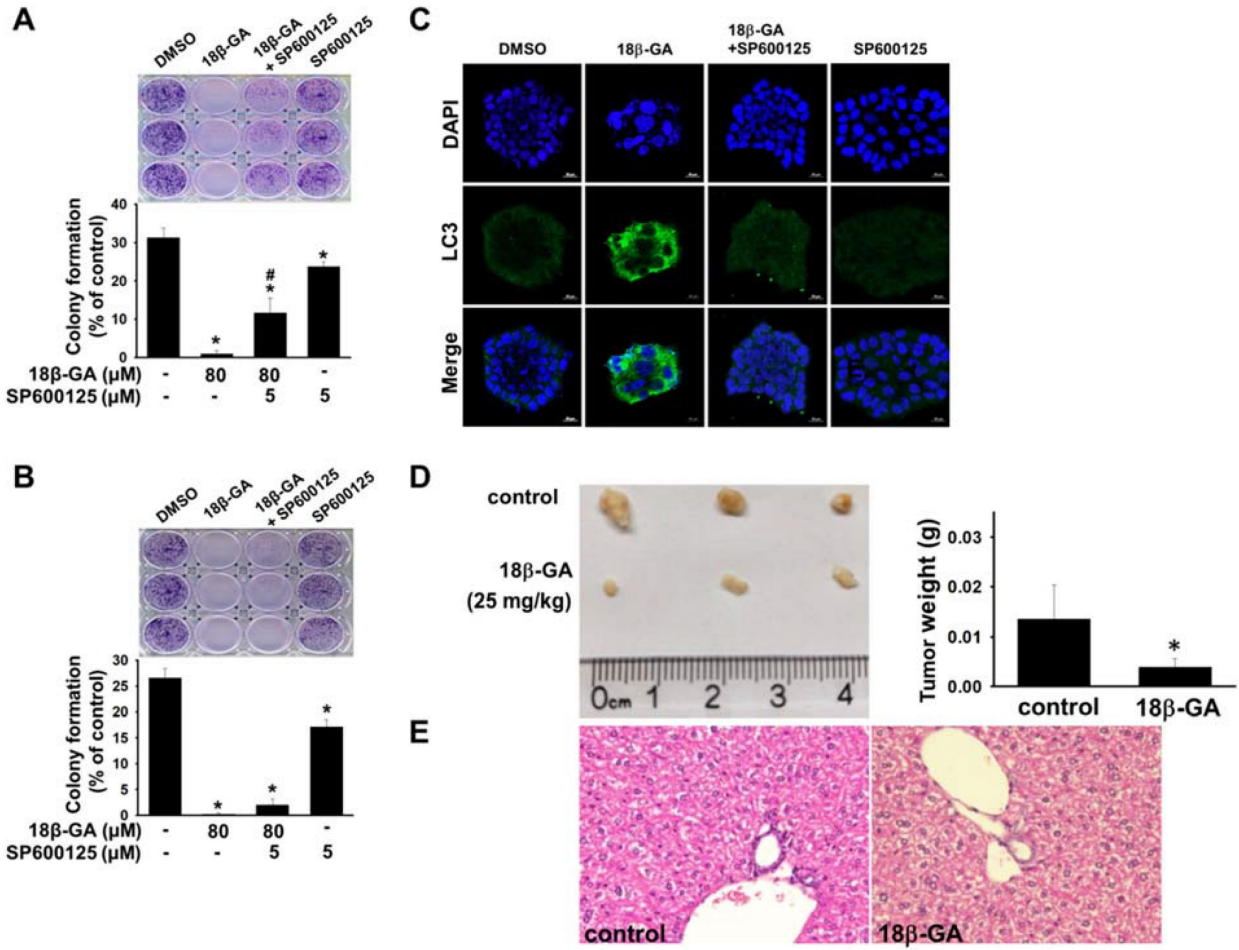
**18β-GA suppressed long-term clonogenic survival and tumor growth.** MCF-7 (A) and T-47D (B) cells were treated with 80-μM 18β-GA in the presence or absence of JNK inhibitor (5-μM) for 6-15 days. Visible colonies were stained using crystal violet. The control group contained 0.1% DMSO. Data are shown as the mean ± SD from three independent experiments. Significance was analyzed using one-way ANOVA followed by the Bonferroni test (*p* < 0.05). * shows a significant difference compared with the control, and # shows a significant difference compared with the 18β-GA treatment. (C) MCF-7 colonies grown in the presence or absence of JNK inhibitor and 18β-GA were stained using anti-LC3 antibody and DAPI. Images were obtained using confocal microscopy with 20× objective. The scale bar is 20-μm. (D) Representative images of tumors and tumor weights from BALB/c nude mice in control and 18β-GA (25-mg/kg) treatment over 12 days following MCF-7 xenograft (N = 3-4). Significance was analyzed by the independent t-test (*p* < 0.05). * indicates a significant difference compared with the control. (E) Representative hematoxylin and eosin staining of liver from BALB/c nude mice bearing MCF-7 xenografts treated with vehicle, and 18β-GA (25-mg/kg). Images were acquired using light microscopy with 40× objective.

Upregulation of JNK mediates the induction of autophagy 18, 19. It was investigated whether JNK is involved in 18β-GA-modulated autophagy using Western blotting. Figs. 5, 6A and 6B indicate that 18β-GA increased the phosphorylation level of JNK. Rapamycin-induced autophagy also increased apoptosis and phosphorylation level of JNK (Fig. 5). Pre-treatment with SP600125 (JNK inhibitor) reduced LC3-II and cleaved PARP, which were induced by 18β-GA. The apoptosis proportion caused by 18β-GA and SP600125 in MCF-7 and T-47D cells were simultaneously inhibited (Fig. 6). These results proposed that p-JNK is the common pathway to regulate apoptosis and autophagy in MCF-7 and T-47D cells. We further examined p-JNK in long-term clonogenic survival (Figs. 7A and 7B). SP600125 suppressed 18β-GA induced LC3-II accumulation (Fig. 7C), whereas it reversed the numbers of MCF-7 and T-47D colony formation (Figs. 7A and 7B).

### 18β-GA inhibited tumor growth

To confirm the antitumor effect of 18β-GA *in vivo,* we subcutaneously inoculated MCF-7 cancer cells to conduct a mouse xenograft experiment. It was found that i.p. injection of 18β-GA at a dose of 25-mg/kg inhibited tumor growth (Fig. 7D). The body weight of each mouse showed no significant alteration during the examination period (data not shown). Cell morphology of the liver in the 18β-GA treatment group was similar to that of the control (Fig. 7E).

## Discussion

The role of autophagy in breast cancer is complicated, and its possible role as cell death or a cell survival promoter still needs further investigation 16, 17. In this study, it was shown that 18β-GA reduced the cell viability of MCF-7 and T-47D through cell cycle arrest, apoptosis, LC3-II accumulation, or blocking autophagic flux. The induction of autophagy by rapamycin can enhance 18β-GA-mediated apoptosis, whereas inhibition of autophagy with 3-MA can reverse 18β-GA-mediated apoptosis. Activated JNK is involved in 18β-GA-induced apoptosis and LC3-II accumulation. The JNK inhibitor (SP600125) suppressed apoptosis, LC3-II accumulation and rescued the colony formation of MCF-7 and T-47D cells. 18β-GA can further inhibit tumor growth in BALB/c nude mice bearing MCF-7 xenografts. All data suggest that 18β-GA-modulated autophagy is cytotoxic by promoting apoptosis and JNK activation.

The antiproliferative activities of 18β-GA have been tested in different cell lines 10. Our study showed that 100-μM 18β-GA induced G0/G1 phase arrest in MCF-7 and T-47D cells at 24 h (Fig. 2). 18β-GA (≤25-μM) causes G0/G1 cell cycle arrest in non-small-cell lung cancer cells without apoptosis 20. Satomi* et al.* reported that a 50% inhibitory dose of GA arrested the cell cycle in the G0/G1 phase, while a higher dose can induce apoptosis 21. Combining these results, it was proposed that the dose of 18β-GA notably influences the inhibition type of proliferation in various cancer cells.

Induction of apoptosis has been identified as a vital therapeutic strategy for treating cancer cells. This study found that a higher concentration of 18β-GA (150-μM) induced caspase-dependent apoptosis in MCF-7 and T-47D cells (Fig. 3), which supported the previous discovery that 18β-GA can induce apoptosis in MCF-7 cells and hepatocellular carcinoma HepG2 12, 21. Also, 18β-GA-induced apoptosis was not entirely prevented by pan-caspase inhibitor action in MCF-7 and T-47D cells (Fig. 3). Moreover, induction of autophagy resulting in pro-death or pro-survival is considered a treatment strategy for breast cancer. It was shown that 18β-GA upregulated LC3-II conversion in T-47D cells, and LC3-II accumulation blocked autophagic flux in MCF-7 cells (Fig. 4). Combined treatment with rapamycin enhanced the sensitization of 18β-GA-mediated apoptosis (Fig. 5), showing that autophagy-associated apoptosis was efficient induction in such combined treatment. Inhibition of endogenous autophagy by 3-MA reversed 18β-GA-mediated apoptosis (Fig. 5). These results show that 18β-GA-modulated autophagy exerts anti-cancer activity through apoptosis promotion in breast cancer cells (MCF-7 and T-47D). Previous studies reveal that autophagy inhibition enhances 18β-GA-induced apoptosis in hepatocellular carcinoma and human sarcoma cells, suggesting that 18β-GA-triggered autophagy is protective for these two cell types 13-15. Altogether, interactions between autophagy and apoptosis under 18β-GA treatment are cell type dependent and it is unique to find that 18β-GA induced autophagy promotes apoptosis in MCF-7 and T-47D cells.

Also, 18β-GA-induced apoptosis is partially mediated by the JNK signal pathway in pituitary adenoma 22. The JNK pathway is also involved in enhancing 18β-GA-induced apoptosis by blocking autophagy in human sarcoma cells 15. In this study, our results showed that 18β-GA can induce apoptosis and LC3-II accumulation or arrest autophagic flux in breast cancer cells (Figs. 3 and 4). Combined treatment using a JNK inhibitor decreased 18β-GA-mediated apoptosis and accumulation of LC3-II (Fig. 6), suggesting that p-JNK is a common regulator between 18β-GA-induced apoptosis and modulated autophagy in MCF-7 and T-47D cells. Although there are bioenergetics variations between these two cell lines 23, autophagy and apoptosis modulated by 18β-GA in our study are indicated in the common JNK pathway. 18β-GA modulated autophagy involved in long-term survival of breast cancer cells is also confirmed through p-JNK (Figs. 7A-C). Sharma *et al*. showed that 18β-GA-induced apoptosis in breast carcinoma MCF-7 occurs through the Akt/FOXO3a pathway 12. Whether the activation of JNK contributes to the Akt/FOXO3a pathway in the enhancement of 18β-GA-induced apoptosis by blocking autophagy is worth further investigation.

18β-GA can displace 17β-[3H]-estradiol from sex hormone-binding globulin but not from the ER 24. MCF-7, T-47D (ER+) and MDA-MB-231 (ER-) response to 18β-GA are not dependent on estrogen receptor (Fig. S1 and S2). The concentration of 18β-GA used in the study is 80-150-μM. Studies show that the toxicity of 18β-GA in animals and humans is very low 25, 26. In mice, the acute intraperitoneal LD(50) was 308-mg/kg and the oral LD(50) was >610-mg/kg 25. 18β-GA (25-mg/kg) already expressed the inhibition of tumor growth in BALB/c nude mice (Fig. 7D). In humans, its pharmacological safety has been proven at a non-toxic consumption of up to 1.5-g/day, and the plasma peak concentration can be over 200-μM 26. The low toxicity of 18β-GA in humans benefits its development as an anti-cancer therapy for breast cancer treatment. Moreover, it is worth investigating its more effective derivatives that can modulate autophagy to promote apoptosis.

## Conclusions

In this study, it was shown that a lower concentration of 18β-GA causes G0/G1-phase arrest, and a higher concentration of 18β-GA inhibits cell survival of luminal A breast cancer cells by inducing apoptosis and LC3-II accumulation. Treatment using an autophagy initiator can increase 18β-GA-mediated apoptosis and JNK activation in MCF-7 and T-47D cells, in contrast, which can be reversed by an autophagy inhibitor. Additionally, the inhibition of JNK attenuated 18β-GA-mediated apoptosis and LC3-II accumulation in long-term clonogenic survival. All data suggest that 18β-GA modulated autophagy is cytotoxic through apoptosis promotion and JNK activation. Modulation of 18β-GA-mediated autophagy may apply to a cytotoxic strategy to treat luminal A breast cancer with hormone therapy resistance.

## Supplementary Material

Supplementary figures.

## Abbreviations

18β-GA: 18β-glycyrrhetinic acid
3-MA: 3-methyladenine
JNK: Jun-amino-terminal kinase
ER: estrogen receptor
PR: progesterone receptor
HER2: human epidermal growth receptor 2
FBS: fetal bovine serum
PARP: poly (ADP-ribose) polymerase
LC3: microtubule-associated protein 1 light chain 3
GAPDH: glyceraldehyde-3-phosphate dehydrogenase
RT: room temperature
PBS: phosphate-buffered saline
PI: propidium iodide
FITC: fluorescein isothiocynate
RFP: red fluorescent protein
GFP: green fluorescent protein
i.p.: intraperitoneal
SD: standard deviation
CQ: chloroquine
