# Supplementary material for: 18β-glycyrrhetinic Acid Modulated Autophagy is Cytotoxic to Breast Cancer Cells
Source: Int J Med Sci. 2023 Feb 13;20(4):444–54. doi: 10.7150/ijms.80302 (PMC10087636; doi:10.7150/ijms.80302)
Supplement: Supplementary file 1 — Supplementary figures. [file ijmsv20p0444s1.pdf]

Supplementary Figures

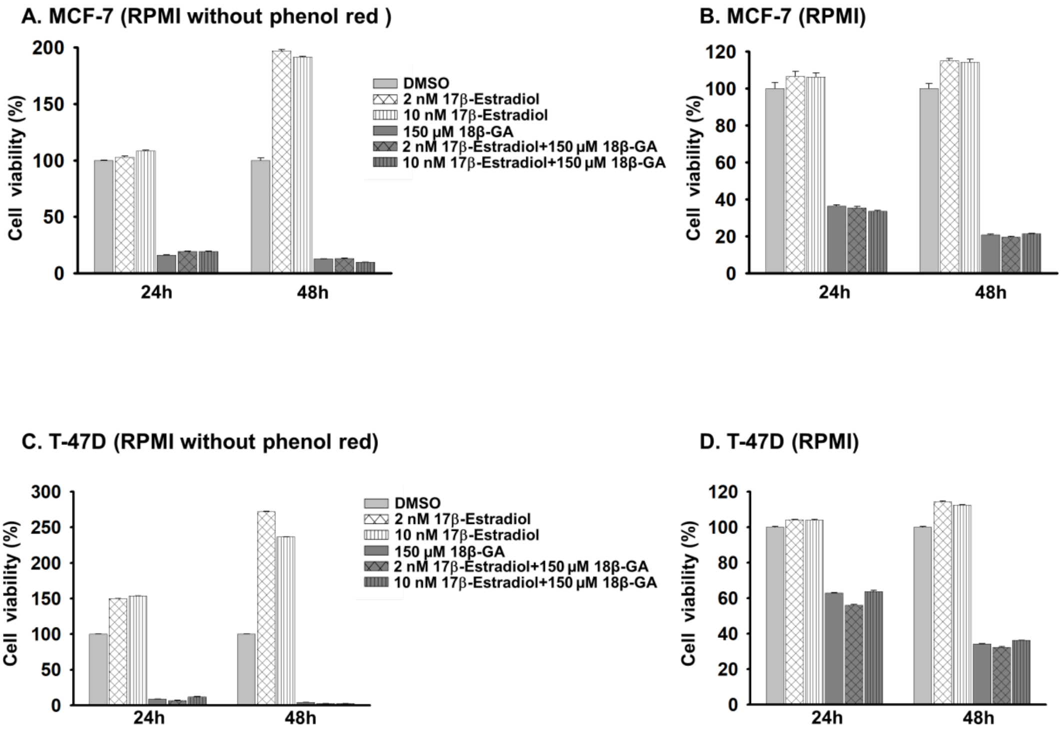

Figure S1: Effect of estrogen on proliferation of 18β-GA treated luminal A breast cancer cells. MCF-7 (A, B) and T-47D (C, D) cells were treated using 150-µM of 18β-GA combined with 17β-estradiol in phenol-red free medium containing 5% -charcoal dextran stripped FBS and normal RPMI culture medium for 24 and 48 h. Cell viability was detected using the CCK-8 assay. The data was the represented results of 2-3 repeats.

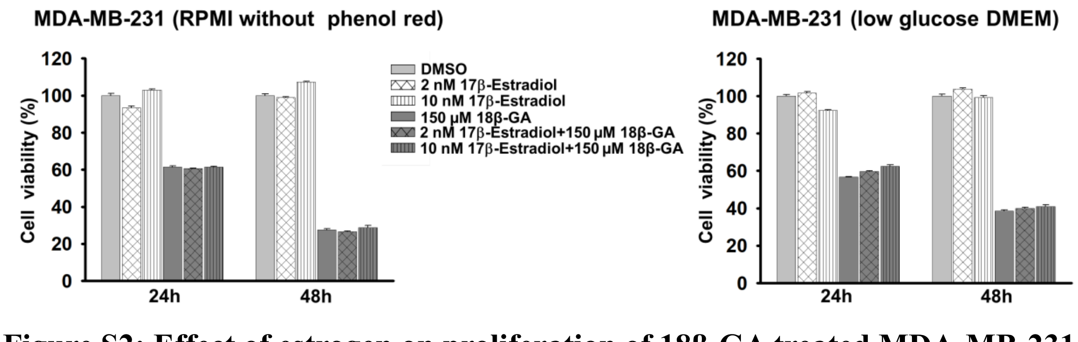

Figure S2: Effect of estrogen on proliferation of 18β-GA treated MDA-MB-231 cells. MDA-MB-231 cells were treated using 150-µM of 18β-GA combined with 17β-estradiol in phenol-red free medium containing 5% -charcoal dextran stripped FBS and normal RPMI culture medium for 24 and 48 h. Cell viability was detected using the CCK-8 assay.
